# A Few Remarks on Acute Laryngitis with Illustrative Cases

**Published:** 1855-06

**Authors:** Thomas F. Rochester

**Affiliations:** Professor of Theory and Practice of Medicine in Buffalo Medical College


ART. II.—*A few Remarks on Acute Laryngitis with Illustrative Cases.* By THOMAS F. ROCHESTER, M. D., Professor of Theory and Practice of Medicine in Buffalo Medical College.

The relative infrequency of Acute Laryngitis, as compared with the other inflammatory affections of the air passages, is justly esteemed a most providential exemption from disease. For as surely as inflammation of a severe grade attacks the voice seat, so surely, unless promptly recognized and readily arrested, does it lead to a rapid and fatal termination. As the disorder is seen but rarely, it seems scarcely possible to urge too forcibly the importance of distinguishing between the various modes of attack, of determining whether it be simple or complicated, and if the latter, of deciding upon the nature of the complication. Spurious or simulated Laryngitis is also to be borne in mind. The sex of the patient will often reveal this at once, *(hysteria morborum omnium simulator,)* as well as the knowledge of the fact, that males are more frequently than females the subjects of the inflammatory seizure.

It is proposed in this communication, to submit to the readers of the Journal, a report of a few cases that have recently been presented to the observation of the writer, and it is hoped that if no new ideas of either pathology or treatment are advanced, that at least a candid statement of facts may not be without interest or value.

CASE I. Miss L—— aged 17 yrs., native of Buffalo, person well developed, health vigorous, menstruated at 15. On the evening of Sept. 13th,

1854, she was overtaken by a rain storm and was much wet; on reaching home, she changed her garments and felt none the worse from the exposure. Retired about 10 P. M. quite well. Awoke at 3 A. M., feeling hot and thirsty, pain in back and limbs, throat sore and voice hoarse, cough slight and without expectoration. Sept. 14th, 9 A. M., I was called to see her. I found her quite anxious and excited, pulse 95, full and hard, tongue slightly coated, bowels confined, surface of body hot and dry, eyes bright, cheeks flushed, voice husky, cough hoarse, spasmodic and frequent, respiration hurried and incomplete. Fauces intensely red but no tonsilitis; I could not obtain a view of the epiglottis; examination was however made freely with the finger, and detected a constantly upright position of the epiglottis, but without positive induration or œdema. No pain was experienced on pressure from without, except over the thyroid cartilage; *there* it was decided, and to the same locality was also referred pain in deglutition; all attempts to drink induced cough, a portion of the fluid probably passing into the glottis, on account of the immobility of its protective valve. Physical exploration of the chest detected insufficient inspiration, and consequent slight pulmonary inflation—there was great heaving and shoulder raising, but no full, deep inspiration; every effort at this was spasmodically arrested. Diagnosis—Acute uncomplicated Laryngitis.

I directed the feet to be soaked in a hot mustard bath, and three Swedish leeches to be applied along the trachea, for I fancy the objection to their use in such cases, as urged by Watson, to be entirely theoretical and quite groundless—but admitting that serous infiltration of the cellular tissue may result from the leeching, surely acupuncture will at once relieve it. After the foot bath I prescribed ol. Ricini ℥ss, succi Limonis ʒij, to be taken at one dose. This formula is, I believe, much used at the Massachusetts General Hospital; the lemon juice covers the taste of the oil and at the same time increases its cathartic action. I also prescribed—Ant. et Pot. Tart. grs ij. Morphiæ Muriatis gr. iss. Syr. Limonis ℥jss. Dose $\frac{1}{2}$ to 1 teaspoonful, to be given sufficiently often to maintain nausea but to avoid emesis.

2 P. M. Patient much relieved; the bowels have moved twice freely; the blood is still oozing from the leech-bites; the tartar emetic produces very slight nausea; pulse 90 and less hard; countenance still anxious; cough has almost ceased; respiration less jerking and irregular; voice remains husky and unchanged; pain diminished both in swallowing and on pressure. Tartar emetic and morphine to be continued; hot poultice of bran and hops to be applied to throat.

9 P. M. Patient about the same as at 2 P. M. Has had four prolonged spasmodic attacks of coughing during the evening, but unattended with expectoration. I directed the stimulating pediluvium to be repeated, and the vapor of warm water to be inhaled if, on trial, it was found to relieve the spasmodic coughing; discontinue the Tartar emetic and to take Pul. Ipecac. comp. grs. x every four hours.

Sept. 15, 9 A. M. Patient has slept well; has taken but two of the Dovers Powders—found the inhalation of the warm vapor very grateful. Pulse 80 and soft. No pain in swallowing and scarcely a perceptible amount on pressure; cough and voice both slightly husky. Respiration unembarrassed; countenance cheerful; the redness of the fauces diminished. *No serous infiltration from the leeching.* No further general medication seemed necessary, but I judged that topical applications would *now* be of service; the fauces and rima glottidis were accordingly lightly touched with a sponge moistened with a solution of Nitrate of silver, grs. xxx to ℥j of distilled water. This produced coughing, followed by mucous expectoration, an effected a decided and instantaneous improvement in the quality of the voice. The sol. of Nitrate of silver was applied every morning for three successive days, and Miss L—— was discharged, quite well. It may be asked, why was not this topical treatment resorted to at the commencement of the attack? With respectful deference to those of an experience more extended than my own, I would answer, that as a sedative and antiphlogistic, I have not found the Nitrate of silver to sustain the favorable reports of its advocates; on the contrary, in several cases of acute Tonsilitis, in which I applied it, it certainly increased the inflammation, and in two instances great œdema of the uvula immediately followed its use. I have seen many cases of tonsilitis at once arrested by the lunar caustic, but I think I have seen more failures than successes. In several instances it aggravated the inflammation, in two it produced positive œdema. When it does afford relief, it is so speedy and so effectual, that I think it should always be tried, where it can be used with safety, as in Pharyngitis, Tonsilitis, and perhaps membranous croup. In the two former affections aggravation of the inflammation would scarcely ever place life in jeopardy, but in Laryngitis, attended with high febrile movement, and with unmistakable evidence of intense local excitement, the chances of the production or of the increase of œdema are certainly equal to those of preventing or of allaying it. In Laryngitis accompanied with secretion, the employment of the Nitrate of silver, as a stimulant and alterative, does not seem to be either dangerous or objectionable, and as a stimulant, it is doubtless a valuable agent in restoring tone and vigor, in cases such as Miss L—'s, when the inflammation is subdued.

Case II. Patrick Flannagan, Irish, laborer, 43 years old, admitted to the Hospital of the Sisters of Charity November 1st, 1854. Disease, Typhus Fever. The attack was of the most grave character. The eruption general, copious, and of a livid aspect, with large vibices upon the lower extremity. His treatment was from the first sustaining. About 3 A. M., Nov. 29th, he was suddenly attacked with great dyspnœa. This was very naturally supposed by the nurse to depend upon an increase of the Bronchitis which accompanied the Typhus Fever. The house physician, Dr. Duforburg, on making his morning round, suspected a new complication, viz. Laryngitis; his surmise proved correct. On account of the dull, heavy and prostrate condition of the man, no very close examination could be made. Dr. D. suspected Laryngitis from the noisy and labored respiration, and from the excessive lividity of the surface. On making my usual morning visit at 11 A. M., I found Flannagan moribund. The symptoms at this time were rather those of coma than of apnea. Autopsy—8 hours after death. There were the usual evidences of blood poisoning and of glandular and visceral engorgement, as well as the diagnostic absence of enlargement or ulceration of Peyer's plates. Dr. Duforburg's clever diagnosis was also verified; the chink of the glottis was closed completely by swelling of the mucous membrane and by serous infiltration in the sub-mucous tissue. This case is reported as evidence of a complication of Typhus, that is unusual. Had the Laryngitis been recognized at the moment of its access, it is not probable that any remedial measures would have been of avail. Tracheotomy might have prolonged, it would not have preserved the life of this patient.

Case III. John G., for several years an inmate of the Hospital, and under treatment for Phthisis Pulmonalis, died in Nov., 1853. This poor man during the last year of his life suffered from three distinct attacks of Laryngitis. They were each attended with aphonia, pain on pressure over the pomum Adami, and almost entire inability to swallow, as well as severe and alarming paroxysms of cough and dyspnœa. Had not previous examinations demonstrated the disease of the lungs, their condition might well have been overlooked, so violent and so absorbing were the laryngeal attacks. These latter, although obstinate, yielded to the administration of Quinine, anodynes, and the inhalation of warm vapor. A ropy, glairy mucus was always secreted by the inflamed mucous membrane, and contrary to what might have been expected, the dyspnœa was always in proportion to the quantity of the secretion. I accounted for this upon one of two suppositions: either the secretion was so acrid that by local irritation it induced spasm, or it was so abundant and tenacious, that it filled up the glottis and trachea, and from its glutinous nature was with difficulty dislodged and expectorated. In this instance the local application of the Nitrate of silver, several times afforded temporary relief. G. did not die from either suffocation or coma, but from asthenia. The autopsy revealed cavities in the apices of the lungs, and crude and softening tubercles throughout their entire extent. The epiglottis was very much enlarged and indurated, its laryngeal surface was deeply fissured, and at the same time studded with numerous elevations of a fibrous character, varying in size from a line to 1-12 of a line in diameter. The same appearances, though less marked, were seen throughout the lining of the Larynx; its cavity however was rather dilated than diminished. May not this be cited as an instance of sub-acute Laryngitis occasionally assuming an acute form, and producing, as the result of repeated attacks, dilatation in place of diminution of cavity.

Case IV. Wm. Muller, German, age 38 years, barber by trade, admitted to the Hospital Jan. 16th, 1855, is much emaciated and presents the appearance of a person in advanced Phthisis; has had cough for the last three years, especially in winter; has never expectorated much; has never had hæmoptysis; is subject to diarrhea; within the last fortnight has had nightsweats; took a severe cold three weeks since, and has been constantly growing worse. Present condition: voice whispering and almost entirely labial; cough incessant and brassy (rauca); expectoration abundant, tenacious and glairy; swallowing produces pain and cough; marked tenderness over the thyroid cartilage; pulse frequent and feeble; surface pale, cold and clammy; lips and nails livid. Fauces pale; epiglottis easily brought into view; its color contrasts strongly with the membrane of the fauces, being of a bright and vivid red; it is enlarged, erect and almost immovable. The attempt to pass the finger into the cavity of the glottis was frustrated by the spasms, induced by the touch. Physical exploration of the chest afforded, on percussion, a fair amount of resonance, and no marked comparative inequality; auscultation discovered a faint respiratory murmur, prolonged expiration with mucous and sub-crepitant râles. The general appearance of the patient, his cough of three years duration, and the physical examination as far as it could be made with the patient breathing in a noisy and labored manner, all indicated Phthisis, yet the diagnosis was not positive as to this disease. As to the Laryngitis, there was no question. Muller's enfeebled condition forbade the employment of antimony, or of even local depletion. I directed a blister to be applied to the upper sternal region, and one of the following powders to be taken every four hours. ℞. Hydrarg. chlor. mite gr. ss.; Pul. Ipecac. comp. grs. v.; Quiniæ Sulph. grs. ij.

Jan. 17, 11 A. M. To my great surprise I found Muller up and walking about, his general appearance much improved; he is in fine spirits and thinks that he will be quite well in a month; his voice remains labial, his cough hoarse, and the expectoration glairy and abundant; his breathing, however, is unembarrassed, although hurried. On inspecting the fauces, I found the epiglottis tumid and erect, but it had lost its bright florid hue. I carried my finger easily into the chink of the glottis without inducing spasm. Treatment—the blistered surface to be dressed with Unguentum Belladonnæ, otherwise, continued as on yesterday.

Jan. 18. Patient doing well, except that his bowels are moving too freely. Discontinue the calomel, and continue the quinine and Dover's Powder.

Jan. 20. Made another physical examination of the chest; my opinion of presence of softening tubercles confirmed. Continue Dover's Powder and quinine, and give also, a teaspoonful of cod-liver oil, after each meal.

Jan. 23. A recurrence of the painful and laborious breathing, and all the other symptoms of laryngeal disease that are described as having been present on the 16th. These were soon succeeded by coma, which proved fatal about midnight. Autopsy 16 hours after death. Lungs filled with miliary tubercles, softening tubercles, but no cavities. The appearance of the larynx corresponded with singular minutiæ to that of Case III. The membranes of the epiglottis and arytenoid cartilages were thickened, and studded with elevations, but the cavity of the glottis was not diminished. The specimens are deposited in the Museum of the Medical department of the University of Buffalo.

Case V. Thomas Gibbons, laborer, native of Ireland, age 23 years, had been for some weeks an inmate of the Hospital, under treatment for an unhealthy ulcer upon the foot. On the morning of March 28th, 1855, as I was passing accidentally through one of the surgical wards, my attention was attracted by the hurried breathing and anxious countenance of one of its inmates. As soon as the man caught my eye he *beckoned* me to his beside. This was truly a "gesture of disease." The attentive and efficient *interne*, Dr. I. N. Brown, informed me that Gibbons had recovered from his ulcer, but had been suffering for twenty-four hours from tonsilitis, induced by careless exposure to cold,—that his present manner of breathing had come on since he had last seen him, not two hours before. I acceded to the imploring look and gesture of the sufferer, and in the absence of Prof. Hamilton, in whose ward he was, took the liberty of examining him. The evidences of tonsilitis were decided, although the attack was not severe. The suspicion of laryngitis had crossed my mind, from the manner of breathing, from the anxious expression and from the gesture; the signs however were not positive. The voice was husky, but the cough was slight; there was very little pain on pressure over the pomum Adami; there was much more in the left posterior sub-maxillary region, and to this point was referred all difficulty in swallowing. The countenance was flushed, the temperature of the surface was somewhat elevated, but the pulse beat but 80. Prof. Hamilton coming in at this time I directed his attention to the patient. He had not seen him in this illness, and at once transferred him to the medical department to which he properly belonged. Continuing my examination, I endeavored to get a view of the epiglottis, but was unable to do so for a reason that will appear hereafter. I could not divest my mind of the idea of laryngitis, but I was inclined to the opinion that if present at all it was to a very light degree. The man was Irish, and consequently naturally inclined to represent his condition as worse than it really was; he was weak from a depraved state of his system, and nervous from long confinement in a ward filled with sick people. All these circumstances, with the unaccelerated pulse, induced me to conclude that it was, after all, only tonsilitis. To err upon the safe side, however, I directed four leeches to be applied over the trachea, to be followed by a hot poultice, and 10 grs. of Dover's powder, to be given every four hours. Feeling somewhat anxious as to the correctness of my ultimate diagnosis, I revisited Gibbons at 5 P. M. Every doubt was now dispelled; the man was breathing quietly; his pain and cough had ceased, and there was not a trace of anxiety upon his countenance; he expressed himself as being very comfortable, and I left him, simply directing a Dover's powder at bedtime. At 11 P. M. I was summoned in great haste to the Hospital. The messenger did not know on whose account, and on arriving I was much surprised to find that at 10½ P. M., Gibbons, who had been coughing for an hour or more, was seen to rise up in his bed, struggling for breath—he was unable to speak; he dashed wildly about the room with his arms uplifted and gasping, and at length sank, completely exhausted, upon the floor. The paroxysm over, he breathed in a broken, gasping manner, and coughed and spit incessantly. When at a considerable distance from his bed, in fact in the hall of the floor below his ward, I could hear his noisy, struggling breathing; I found him sitting half upright in bed, his countenance pale and anxious; extremities cold and nails livid; his pulse very feeble, but not over 90; he was spitting up large quantities of mucus deeply tinged with blood; he spoke in a hoarse whisper and begged me to relieve him, "for he was strangling." I thrust my finger into the fauces, and found the epiglottis firmly *contracted* upon the rima glottidis. I could neither lift it or move it from side to side. This accounts for my not seeing it in my examination in the morning. The secretion being so abundant, and at the same time so bloody, I was in hopes that nature would soon afford relief, and I sat down, not expecting to be obliged to interfere, and endeavored to tranquillize the patient, for he was exceedingly alarmed. In a few moments, however, unmistakable indications of another severe paroxysm were evident—there was no room for doubt, and without an instant's delay I opened the crico-thyroid space with a very neat instrument belonging to my friend, Dr. Nelson, (a curved trochar adjusted to a double canula). In rushed the air, and poor Gibbons, who had been terribly frightened at the idea of a hole in his throat, drew a long full breath, and with a sigh expressive of infinite satisfaction, sank almost at once into a quiet slumber. From this he was aroused by a paroxysm of coughing, discharging through the tube and by the mouth a large amount of bloody mucus; there was very little hæmorrhage from the incision, and no blood passed into the trachea from the wound; the bloody expectoration was of the same character that it had been before the trachea was opened. The advantage of a double over a single canula was now very apparent. From the large amount of secretion, the tube filled up rapidly, and although the efforts of the patient were generally sufficient to clear it, yet on the slightest obstruction the inner tube was at once taken out, cleaned and replaced, the outer one keeping its position, and affording, meanwhile, free passage to both air and fluid. Directed a little brandy and water to be given occasionally, and in case of pain or restlessness, 10 grs. of Dover's powder.

Thursday, March 29th, 10½ A. M. Gibbons has not slept much, but has passed a quiet and comfortable night. Swallowing is attended with some difficulty; no fluid, however, passes into the trachea; the fauces are less red than yesterday, and the epiglottis remains contracted upon the rima glottidis; pulse 85; surface moist; nails no longer livid; expectoration abundant, and tinged with blood; cough paroxysmal, and at times severe. Just before my visit the string retaining the outer canula had broken, and the instrument had been thrown out. I replaced it, and secured it by a stronger cord. The eyes of trachea tubes should never be pierced for small cords; they should be sufficiently wide to admit a strong tape; there is do danger of chafing or breaking this, and besides, it may be drawn tighter than the cord, as its flat surface will not cut the skin or make great pressure upon the cervical veins.

I prescribed nutritious diet, and one grain of quinine with five grs. of Dover's Powder, every four hours.

5 P. M. Patient doing very well. Can speak quite distinctly when the orifice of the tube is closed; the epiglottis can be felt in an erect position; it does not, however, act as a valve, for when fluids are swallowed, a portion passes into the trachea and is thrown out through the tube. Treatment continued.

Friday, March 30th, 10 A. M. Patient breathes easily, and has but little cough. Expectoration diminished and muco-purulent; swallows without pain, but fluids still pass into the trachea and out through the tube. Changed the tube for another double canula of larger size; to effect this it was necessary to enlarge the wound in the integument, but not in the crico-thyroid space. Patient says he breathes more easily through the larger tube, and that he feels more comfortable with it than the other. 5 P. M. Patient feels well, and swallows without any escape of fluid through the tube. He is sweating, however, profusely, and his pulse is 100, and feeble. Prescribe, in place of quinine and Dover's Powder, quinine and sulphuric acid, with half a grain morphine, at night if necessary—the bowels being confined—to be moved with a mild enema.

Saturday, March 21st, 10½ A. M. Has slept well; countenance cheerful; pulse 85; cough very slight; no pain or soreness about the larynx. Has a good appetite, and is anxious to leave his bed; has permission to do so.

Sunday, April 1st, 5 P. M. I found Gibbons up and dressed. The laryngitis appears to have entirely subsided.

Monday, April 2d, 10 A. M. I removed the tube, leaving the wound uncovered.

5 P. M. Patient breathes well, and expectorates very little; none of the fluid escapes through the wound, which is contracting rapidly.

Saturday, April 7th. Gibbons left the Hospital.

Tuesday, April 17th. Gibbons came to see me at my office, and reported himself perfectly well.

There are several points of interest in this case:

*First.* The supervention of laryngitis, tonsilitis being the primary disease, a sequence or accident that is very unusual, although more apt to ensue when there is no œdema of the uvula, as in this instance, than when that is present.

*Second.* The mildness of the attack, its apparent subsidence, its very sudden and equally violent return.

*Third.* The peculiar position of the epiglottis, and the tenacity with which it was held down by the combined force of spasm and inflammation, for that the latter was present, and in a high degree, is abundantly evinced by the character of the expectoration; that both the inflammation and spasm should not have been relieved by the free mucous and bloody discharge, appears singular.

*Lastly.* The rapid subsidence of the laryngeal inflammation, no other local treatment being resorted to than laryngotomy.

As to the treatment adopted, a few remarks may not be out of place.

The depraved condition of the man's system, placed out of question the employment of general blood-letting, or the use of mercury or antimony. Had the health of the patient been vigorous, the onset of the attack was so mild, and the diagnosis as respects laryngitis so doubtful, resortt o powerful measures would hardly have been warranted.

The fixed and anomalous position of the epiglottis rendered Buck's method of scarification impossible; that this position did not depend upon spasmodic irritation, temporarily induced by the introduction of the finger, is shown by its (the epiglottis), absence from view on the first examination, and by the non-passage of fluids into the trachea until after the inflammation began to subside, and their subsequent escape into it, when the epiglottis became erect, but was for a time incapable of performing its valvular office. It is fair to infer that there was swelling of the whole laryngeal membrane,—to how great an extent, it was impossible to judge, but that the principal occlusion of the glottis was occasioned by the epiglottis, resting like a foreign body in the chink, certainly seems evident. Looking upon the laryngeal inflammation in this instance as secondary, and proceeding by direct extension from the tonsils, just as we see the false membrane extending in many cases of croup, the application of the nitrate of silver, when tonsilitis alone was present, might have limited the inflammation and prevented the laryngeal complication. This opinion does not in the least conflict with the one expressed about the employment of nitrate of silver in remarks under case I.

One remark more with reference to the treatment of Gibbons. It will be remembered that on my first visit to him, and when the laryngeal symptoms were very slight, I caused leeches to be applied over the trachea, and apparently with very good results; yet in less than twelve hours after their employment, it was necessary to resort to laryngotomy. I do not cite this as an instance of cause and effect. I think it was not; judging from external appearances, no serous infiltration followed the leeching, yet it would be highly disingenouus in me not to call especial attention to this point, particularly as I have dissented from the idea of danger from leeching.

From an examination of the cases above detailed, it will be seen that all of them, except the first, were in persons of enfeebled condition, and that they could not have borne the active treatment and free depletion that is usually advised. I have in my mind two other cases, both of which occurred in delicate people; hence the inference is drawn that laryngitis of the acute form is more apt to attack the feeble and sickly than the robust and vigorous. Two of the seven had phthisis pulmonalis; they were not cases of laryngeal phthisis,—hence it is also conjectured that phthisis predisposes to laryngitis. As I have had no opportunity of making post-mortem examinations of those who have died from other causes, but who have in the course of their lives had two or more attacks of laryngitis, and yet have not been tubercular, I have no facts to offer to sustain the opinion that *dilitation* of the larynx is the ultimate result of repeated attacks of inflammation of its membranes—the dilatation being purely mechanical, and produced by violent coughing and by severe efforts at inspiration. In the first, third, fourth and fifth cases, the paroxysmal nature of the attacks of dyspnœa is noteworthy; hence the administration of anodynes, antispasmodics, and sometimes of tonics would seem to be indicated. While we remember that inflammation is primarily the cause of the spasm, and therefore mainly direct our efforts to remove the root of the evil, surely we may at the same time combat both source and sequence. Following the general law of disease, all of the cases cited commenced with a night attack.

I have a laughable instance of spurious laryngitis to append: A little after midnight on the 7th of April, I was summoned in great haste to see a lady who was "dying from suffocation." Providing myself with probang, long forceps, et cet., I hurried to the sufferer. As I was ushered into the room, I saw a middle aged female throwing herself about on the floor, in great agony, and breathing in a hoarse and noisy manner. I learned that she had retired about two hours before in good health. She could make no reply, not even in whisper, to my interrogations; her husband said that he thought she fell asleep with something in her mouth. I assisted the lady to a chair, and as she moved to sit upon it, I noticed that her harsh breathing ceased for a moment. Exposing the pharynx to a strong light I saw nothing amiss. I discovered nothing by examination with my finger. I took a long sponge-and-movable-blunt-hook-armed probang in my hand with a view of further exploration. It is an ugly looking weapon to put down a lady's throat, and as I brought it in sight, I was not much surprised to hear her exclaim, "Oh!

sir, you are not going to put that down?" "No, madam, you need have no such fear." She had retired about 10 o'clock, and had fallen asleep with a *gum-ball* in her mouth; on awakening two hours afterwards, she thought that it was sticking in her throat, and that she could neither breathe or speak.

